# ‘They already operated like it was a crisis, because it always has been a crisis’: a qualitative exploration of the response of one homeless service in Scotland to the COVID-19 pandemic

**DOI:** 10.1186/s12954-021-00472-w

**Published:** 2021-03-03

**Authors:** Tessa Parkes, Hannah Carver, Wendy Masterton, Danilo Falzon, Josh Dumbrell, Susan Grant, Iain Wilson

**Affiliations:** 1 Salvation Army Centre for Addictions Services and Research, Faculty of Social Sciences, University of Stirling, Stirling, Scotland; 2 Faculty of Social Sciences, University of Stirling, Stirling, Scotland; 3 The Salvation Army, Homelessness Services Unit, Edinburgh, Scotland

**Keywords:** COVID-19, Pandemic, Homelessness, Substance use, Drugs, Alcohol, Harm reduction, Scotland

## Abstract

**Background:**

The COVID-19 pandemic has necessitated unprecedented changes in the way that services are delivered to individuals experiencing homelessness and problem substance use. Protecting those at high risk of infection/transmission, whilst addressing the multiple health and social needs of this group, is of utmost importance. The aim of this novel qualitative study was to document how one service in Scotland, the Wellbeing Centre run by The Salvation Army, adapted in response.

**Methods:**

Care was taken to identify methods that did not create additional stress at this pressured time. Semi-structured interviews were conducted with Centre clients (*n* = 10, in-person and telephone) and staff (*n* = 5, telephone), and external professionals (*n* = 5, telephone), during April–August 2020. These were audio-recorded, fully transcribed, and analysed using Framework. Service documents were used to enhance contextual understanding. Analysis was informed by theories of psychologically informed environments and enabling environments.

**Results:**

The start of the pandemic was a time of confusion, disruption, and isolation. Centre staff rapidly adapted methods of engagement to provide a range of comprehensive physical and emotional supports, to both existing and new clients, through telephone and online communication and, eventually, socially distanced in-person support. This involved balancing the risks of COVID-19 infection/transmission with the benefits of continuity of support to those highly vulnerable to a range of harms. Whilst the pandemic created many challenges, it also facilitated removal of barriers, particularly concerning provision of harm reduction services which had previously been severely constrained. Clients described the Centre as a ‘lifeline’, providing stability and safety during a period of profound disruption when other services closed their doors. Strong leadership, intensive team working, support/training for staff, a focus on relationships, and active use of client feedback, enabled responsive adaptation to fast-changing demands and the creation of a ‘culture of care’.

**Conclusion:**

This study provides a unique insight into the pandemic by analysing the response of one homeless service during the height of the pandemic. We present a range of implications that have international relevance for those designing policies, and adapting front-line services, to proactively respond to COVID-19 and the continued public health crises of homelessness and drug-related deaths.

## Introduction

### COVID-19 and homelessness

The novel coronavirus 2019, commonly referred to as COVID-19, is a disease of the respiratory system [1]. The disease has now spread to over 150 countries and has been reported on almost all continents [2]. On 11 March 2020 the World Health Organization declared COVID-19 a pandemic, and concern grew quickly due to the rapid spread and levels of severity worldwide [3]. Although everybody is at risk of infection, some individuals are more at risk of ill health from COVID-19 than the rest of the population, due to increased likelihood of severe disease and/or death, or health measures put in place to try to contain the virus which have a detrimental effect on already challenging life situations [4]. Although pre-existing health conditions increase risk, the social determinants of health also make people from marginalised communities, such as people who are homeless, more vulnerable to COVID-19, even without underlying health conditions [5].

Homelessness is a term used to describe people who are without a stable, suitable, permanent home [6], including those who are rough sleeping, residing in hostels or the homes of others, or any other insecure/unsuitable housing. There is a clear association between homelessness and substance use, with many people who experience homelessness using drugs and/or alcohol [7]. The reasons for use are complex, with many people using substances to cope with previous/current violence, trauma, or extremely challenging life circumstances [8]. People who are homeless and use substances are at significant risk of being negatively affected by COVID-19 [2, 9–11]. Although only 16 people in this population group are confirmed to have died from the disease in England and Wales [12], this has largely been attributed to the rapid rehousing of people in private rooms, for example in hotels where they could self-isolate more easily [13]. It has been estimated that there could have been as many as 21,092 infections, 1164 hospital admissions, and 266 deaths, of people experiencing homelessness in England if no action had been taken [14]. In other countries such as the USA, death rates for people experiencing homelessness have been much higher [15].

### COVID-19: substance use and harm reduction

To address the increased risk of harm in the context of COVID-19, there has been a call for rapid changes to substance use services and treatment, in particular, with many changes having been operationalised worldwide with varying success. These have included: ensuring people have access to COVID-19 screening and testing [16]; increases in telehealth for consultations and prescriptions [17–20]; changes from daily pick-up prescriptions of opioid substitution treatment (OST) medications to weekly or monthly where suitable [21, 22]; decreased OST dose supervision [16]; improved access to naloxone [16, 23]; medication delivery [3, 21, 23–25]; increased availability of benzodiazepine maintenance therapy [23]; increased injecting equipment provision (IEP) to address risks of blood-borne virus (BBV) transmission [16]; increased awareness of the need for clean water for injecting [26]; and general guidance about reducing COVID-19 spread in recovery/treatment services [27, 28], reducing harm for people who use drugs in shelter/hostel settings [29], and for drug service providers [30]. Specific harm reduction strategies have also been introduced for people with problem alcohol use, including: access to withdrawal management medications [3]; safer drinking tips [31]; clear guidance for healthcare providers of this client group [32]; and implementation of Managed Alcohol Programmes (MAPs) [33].

In Scotland there have been examples of swift and co-ordinated responses to the pandemic among NHS, third sector, and wider statutory services [34]. Similarly to the rest of the UK, people who were homeless in Scotland were rapidly rehoused in hotels which meant that they were able to self-isolate more easily [35]. Other notable adaptations included: thorough contingency planning for community pharmacy disruption [36]; assertive outreach by medical staff including home visits [34]; increased IEP, for example through postal delivery [37]; rapid access to OST [36]; increased provision via telehealth [34]; and increased naloxone provision [38]. Before COVID-19, services that were not specifically drug treatment services were unable to distribute naloxone. This law was changed by the Lord Advocate in response to the pandemic [39]. Scottish Health Action on Alcohol Problems (SHAAP) also released recommendations for alcohol services and advice for people with problem alcohol use, although this was not specific to people who are homeless [40].

### Aim and theoretical approach of study

While substantial service provision changes have been observed worldwide for people experiencing homelessness and problem substance use, there is a substantial gap in research regarding how those closely involved experienced such changes. This study was undertaken during the pandemic to address this gap by documenting the views and experiences of those involved in providing, using, and working alongside one third sector homeless service in Scotland. The aim was to document how the service changed in response to the pandemic, and associated benefits and risks. The main study research questions are listed below (with the full set provided in Additional file 1):

1. How did clients’ needs change in the early days and weeks of the COVID-19 pandemic?
2. What was already in place in the Wellbeing Centre to meet these needs?
3. What changes and adaptations have been implemented since the start of the COVID-19 pandemic for those experiencing homelessness/risks of homelessness and/or substance dependencies?
4. What opportunities/benefits/challenges/barriers/risks have been presented by COVID-19?

The study was informed by two theoretical approaches: psychologically informed environments (PIEs) [41] and enabling environments [42, 43]. These were drawn upon at the final stages of write up once the inductive element of data analysis had been completed.

PIEs have gained increasing attention in the homelessness sector, and the approach comes from a recognition that people who are homeless have commonly experienced high levels of trauma and deep social exclusion [41, 44]. PIEs advocate for the emotional and psychological needs of the client group to take priority, through low threshold/nonpunitive engagement [45]; adaptations to physical spaces [41, 45]; the creation of organisational cultures that are reflexive and centred around psychological needs [46]; valuing relationships with clients [46]; and fostering a sense of shared ownership [47]. Services informed by PIEs have been shown to improve client outcomes in several ways, including enhanced mental health and wellbeing and reduced involvement with criminal justice and emergency services, and facilitating engagement with health and other care services [48–50].

Duff’s theory of enabling environments [42, 43] is concerned with environmental risks for vulnerable populations: for people who are homeless, and those who use substances, cities are not only spaces of risk and insecurity but can also contain various ‘enabling’ characteristics that are more supportive of health and human development. Duff [43] suggests that enabling environments can only be understood in terms of the enabling resources (social, material, or affective) that operate within a contextual space. For Duff, these enabling resources can operate as a direct result of the design and implementation of specific harm reduction interventions [42] and the unintended enabling resources (created or discovered by people in community spaces) which can indirectly facilitate the success of an intervention.

### The service in focus: The Wellbeing Centre

The Wellbeing Centre (described here as ‘the Centre’) is a drop-in service in Edinburgh, Scotland, for people who are, or at risk of being, affected by homelessness, run by The Salvation Army. The Centre was relaunched in January 2020 to convey the Centre’s priority of supporting their clients holistically, in all aspects of their lives, rather than having a focus on a person’s homelessness per se. It is staffed by a team of nine people (two chaplains, one parish nurse, and two managers 2.5 Full Time Equivalent (FTE), and four support staff who work 2.5 FTE) and, prior to the pandemic, was open every weekday, with 35 people on average attending each day. The Centre runs a drop in facility, café, shower facilities and various groups and social activities. Although not a service specifically for people with problem substance use, many clients use drugs and/or alcohol, and many also experience a range of significant mental and physical health and social challenges. The Centre has a strong harm reduction ethos and, prior to the pandemic, had a visiting IEP service via a mobile van, and experience of having two Peer Navigators based in the service via a research project. The Centre was also in discussion with health partners about running a health clinic in the Centre immediately before the pandemic. Groupwork is also an important feature of the Centre, including a ‘psychosocial programme’ that had run for at least 18 months prior to the pandemic specifically designed to bring together harm reduction, recovery, and wellbeing elements in a low barrier, accessible way.

## Methods

### Approach and ethics

A qualitative exploratory study involving semi-structured interviews and analysis of service documents was conducted between April and August 2020. Care was taken to identify methods that placed the least stress on clients and staff during this challenging time. Ethical approval for the study was granted by University of Stirling’s General University Ethics Panel (GUEP, paper 899) and the Ethics Subgroup of the Research Coordinating Council of The Salvation Army (RCC-EAN200504). Rigorous risk assessments were conducted for face-to-face data collection, as per The Salvation Army and University of Stirling protocols.

### Participant recruitment

Participants were beneficiaries (we use the term clients) of the Centre; service staff and managers; and wider stakeholders who worked closely with the Centre, to ensure that the data collected represented diverse vantage points. Purposive sampling identified individuals based on role/membership of these identified sampling groups and gender, to try to ensure the sample reflected a wide range of views and experiences. Clients were recruited by service staff in several ways. Posters were displayed on Centre walls/doors to provide information about the study, and clients were asked to indicate their interest to staff. Those receiving telephone or online support only (these virtual support mechanisms started during the pandemic and are explained in detail below) were sent an email or text message with details of the study and also asked to indicate their interest in participating to a member of staff. Study details were also relayed by staff verbally to potential participants when people were in the service/attending support group meetings. Contact details of interested participants were passed onto members of the research team to check interest in participating and to arrange interviews. Staff participants were identified by service managers who explained the aims of the study and emphasised that involvement was voluntary, with details of interested participants passed onto researchers. Wider stakeholders were also identified by service managers who emailed individuals with details of the study, and contact details of interested individuals were passed onto researchers. All participants were provided with a participant information sheet and an opportunity to ask questions, and 48-h ‘cool off’ periods were observed.

Informed consent was granted at the beginning of each interview. Written informed consent was provided by staff and stakeholder participants and for face-to-face client interviews. For client telephone interviews, verbal consent was formally provided at the beginning of interviews, with the interviewer reading out the consent form questions individually and the participant saying yes/no to each statement. All interviews were audio recorded with consent and lasted an average of 38 min. The interviews were conducted by two researchers: WM conducted staff and stakeholders interviews and JD conducted client interviews. All interviews were conducted via telephone for staff and stakeholders. Client interviews were either conducted via telephone or in person in the service, to provide choice. In-person interviews were possible as JD was working in the service throughout the lockdown period with required health and safety risk assessments undertaken. Interview schedules differed slightly for each group but covered similar themes (see Additional file 2). After each interview, participants were provided with a debrief sheet which gave further information about the study and support available. Detailed fieldnotes captured researcher reflections to enhance reflexivity [51] and enabled slight changes to be made to interview schedules to enhance clarity.

### Data analysis

Data were transcribed in full and, where relevant, used local Scottish dialect (see Additional file 3 for a glossary) and analysed using Framework [52] in NVivo 12. Framework is suited to policy- and practice-relevant research by providing a structured and transparent approach. The transcripts were split into three separate datasets, one for each participant group, read in full, and then coded line by line by one researcher (DF), with another (HC) reviewing coding. This provided opportunities for discussion on anything that was unclear or could have different interpretations. The research questions guided the data analysis, but data were also coded inductively to allow new ideas to be explored and added to the framework. After coding two transcripts from each participant group (six in total), the initial thematic framework was developed and checked by the wider research team (HC, TP, and WM) and then used to code the remaining transcripts. Finally, each transcript was re-read for completeness to ensure that the final framework was inclusive of all major themes. We decided against using matrices, commonly associated with Framework analysis, because the study findings seemed straightforward to portray without them.

Various documents were provided by service managers to supplement interview data, such as: posters detailing opening times/available services; team meeting minutes; presentations; staff training plans; and information sheets produced on service changes. These were read and analysed (WM) by hand, identifying high-level themes to provide important context when interpreting the interview data and develop a clear timeline for Centre changes (Additional file 4). The timeline provided insight into the types of changes that occurred within the Centre, and exactly when these took place, and helped to understand participants’ experiences of these changes.

## Findings

A total of 20 interviews were conducted with 10 clients, five staff, and five stakeholders. Pseudonyms are used throughout. Table 1 provides participant characteristics.

**Table 1:** Interview participant characteristics

Stakeholders (n = 5) mixed gender—numbers removed to protect identity	
*Third sector organisations*	3
*NHS*	1
*Commissioning*	1
Staff (*n* = 5) mixed gender—numbers removed to protect identity	
Clients (*n* = 10) two women and eight men	

Data are organised into two major thematic categories: firstly, how the Centre reacted to the initial lockdown period and, secondly, how it adapted further as lockdown eased. Secondary themes are used as sub-headers to describe the most significant considerations and challenges encountered, as well as opportunities to enhance the support provided.

### The ‘whirlwind’

#### Confusion and uncertainty, loss and isolation

The initial lockdown period was described as a time of confusion and uncertainty, particularly concerning what support was available for people who were homeless:

There was a couple of weeks where everything changed, and everything stopped. And it wasn’t great in terms of knowing where you could go, what was open, when it was open, how you could get an appointment. All of that was quite hard for a couple of weeks. And support workers wouldn’t necessarily have had the answers either. (Martin, Staff).

Kate (Staff) described the initial stages of the pandemic as a ‘*whirlwind*’ for the Centre. In the weeks leading up to the lockdown (but after the pandemic had been announced) there were discussions amongst the staff team about the need to make changes to the Centre, particularly concerning closure of the café  which provided a well-used community space. Kate described the proposed plans for the Centre to shut completely and her particular concern for clients who were receiving all of their support there. In response to these concerns, Centre management acted proactively, requiring staff to gather client contact details in case of closure to ensure ongoing contact. Although clients were initially told that the Centre would shut, a decision was then made not to do so given it would leave many people unsupported.

There are a lot of individuals that we work with who are not involved with other agencies. And there was a real consensus, a real concern, that these guys were really vulnerable and could slip through the net. It wasn’t just one or two people, there was thirty, forty people.

Owen (Client) described his experience of being released from prison during the early stages of the pandemic, not knowing what support was available, and feeling left to his own ‘*terror’.* His comments provide a powerful example of the kind of concerns staff expressed and the rapidly changing wider service landscape. He had previously mentioned coming to the Centre every day, before his time in prison, to get support:

It started affecting me more when I came out of prison and didnae know what was open or what was shut, and I just felt I was left to my ain, my ain terror. I was left to my ain devices, and I didn’t ken what to do sometimes. I just wanted somebody maybe to speak about my depression or my anxiety and I had really naebody at that time. I didnae know what was available to use, like now I know that I could go online and speak to my CPN [Community Psychiatric Nurse] and that.

As a result of the team’s concerns that the impact of lockdown on client mental health, wellbeing, and substance use was posing a considerable risk, the Centre remained open for one-to-one support on an appointment basis, twice per week initially and increasing to three days a week, as described within the Centre documents. Staff believed that they had the required personal and protective equipment (PPE) to open and operate within guidelines, as Caroline (Staff) described.

That was one of my concerns. That we didn’t have any PPE and I just felt, oh my goodness, I think we should have PPE because some of these clients they come up close, they don’t understand it. So in order to keep us and them safe I was really pleased that after about a week there was PPE available.

While the closure of the café and limited opening hours represented a significant reduction in support, compared to what clients were used to, the client base also grew over lockdown, with levels of support provided to these individuals, and other previously infrequent Centre attenders, actually increasing.

#### Disruption to routines

In addition to feelings of confusion in the early stages, clients also discussed impact on established routines:

My life has changed dramatically like, you know, I was in such a routine. I was training at six o’clock in the morning, every morning, going to work and working and then boxing at night time and it was just my routine. I was on the go all the time and when this happened obviously the gym has shut down and work stopped  and, you know, boxing gym stopped as well like so all my life has gone. (John).

Ross, a client and a daily volunteer at the Centre, described his routine as being ‘smashed to pieces’, and the sudden shock of the planned closure of the Centre, stating that he had not realised the seriousness of the virus until lockdown. For one staff member, Kate, the loss for clients who were volunteering at the Centre before lockdown was particularly significant, as was the loss of ‘being able to spend that time with people’. The community feeling of the Centre was also perceived as especially significant: ‘What people do need is that connection, that community. They want that structure’. Running through interview accounts of disruption to routine and isolation, the loss of the social, drop-in aspect of the Centre looms large. Clients spoke of missing people and viewing the Centre as a community: three described coming to the Centre in these early few days only to not be allowed in or to find the Centre closed. Wayne (Client) described arriving to receive support in the early lockdown period, and not being aware of the lockdown measures: ‘I still thought it was a world away’. Maria (Client) described the sudden nature of the planned Centre closure as hurtful:

Aye, I had a freak out in the building, told everybody that was it, game over, forget it, doors would be shutting, and everybody told me I was being stupid and out of order and then, a week later… boom, doors shut. It wasn’t gradual, it was straightaway. Shut the fricking door, you are not coming back basically. We dinnae want you in the building, we don’t want the responsibility.

However, Jack (Stakeholder) discussed a sense of inevitability about the initial planned closure of the Centre:

I don’t think there was anything different they could have done because they were in the same boat as everybody else. You have to close your doors. Their big fear was they weren’t going to open again.

Samantha (Staff) believed that the plan to initially close the Centre meant that it took a while for knowledge of the new opening times to ‘filter through’ the community. Although the Centre was kept open for one-to-one appointments, the loss of the socialising, comfort and safety provided by the informal ‘drop in’ nature of the Centre, and café, was a significant loss for staff and clients alike. With the café closed, many clients viewed the Centre as having shut completely.

#### Reaching out: staying in touch

Kate (Staff) discussed the initial period following the reduction in services at the Centre as one of crisis intervention, ensuring people had accommodation, food, prescriptions, and were able to isolate safely:

There was a lot of information that staff needed to gather to make sure people were safe. It was a totally different way of working. I wonder if everybody is on prescriptions? Will they be able to access their prescriptions? Do they have a mobile phone? There was a lot of investigating work in the beginning.

The initial reduction of services necessitated a switch to telephone and online support. Whilst staff had made an effort to gather client contact details before the lockdown occurred, they were still left without a means of contacting some. Wayne (Client) described receiving a letter from the Centre informing him of Centre changes and the ongoing support offered: *‘We hope you are okay, please get in touch if you need anything’.* Paula (Staff) reflected on this initial effort to meet needs through crisis telephone support:

We had a list of questions we needed to check with people we were phoning. In some cases it was working well and in other cases it was very difficult because people don’t pick up the phone, people lose their phone. It’s always more difficult to have a chat about those issues over the phone rather than face-to-face.

A major early development was the distribution of smartphones with data to clients who either did not have telephones or who had no means of accessing the Internet. This was made possible through a range of funding sources. The distribution of telephones allowed staff to remain proactively in contact and offer emotional and practical support to clients and allowed the groups, which were a major aspect of the Centre, to occur online. For some clients this was the first time that they had had access to a smartphone. There were initial concerns, external to the service itself, that clients may lose or sell these telephones, but this largely did not materialise. Indeed, provision of smartphones was described as both facilitating communication and showing clients that they were cared about:

Giving people access to phones with unlimited credit and tablets, to be able to communicate. That kind of… not even trust, just saying you deserve this. We want to give this to you so we can stay connected. (Kate, Staff).

Andrew said that receiving twice weekly telephone calls showed that the organisation cared:

Unlike anything else that I’ve ever experienced, to be honest, you know an organisation phoning up checking on your wellbeing twice a week and, you know, it doesn’t really happen.

Frank described how this telephone support helped him maintain his mental health:

Even if it’s just a five minute talk or a twenty minute talk. That cheers me up so much, and my mental health, and it might only just be letting [staff] know what happened. Just letting it out mate, it makes me feel a lot, lot better.

Clients also discussed the online support groups in positive terms, although not all of them engaged this way. Maria, who was less keen on telephone support, described seeing ‘*somebody by the face*’ in the online groups as more comfortable. Others appreciated the online groups because of the more relaxing setting:

I find it quite cool because, in some ways we are, ken like, I find that we are getting to hear each other more. Aye, just because of the situation, we are all sitting in our own wee bit and, ken like, comforts and all the rest of it. (Jacqui).

Owen, who had struggled with mental health problems and substance use during the pandemic, found the groups ‘*a big help*’, a source of mutual support. The long-term benefits of maintaining some form of online access, once the Centre had returned to a more typical service model, were also discussed by clients as particularly beneficial for those who were unwell, or could not attend in person due to poor health.

### Services for social distanced ‘in person’ support

By early May, after the initial period of crisis support and intervention, both staff and clients described a more settled pattern of service provision. Staff were able to provide practical and psychological telephone and online support to clients (as described in the service documents). Not only was the maintenance of services, albeit in adapted form, seen as crucial in supporting the Centre’s existing client base, but also as providing support for individuals who were previously engaged with services which had ceased to operate during the pandemic. Another adaptation, alongside telephone support and online groups, was offering one to one appointments. While some staff commented that keeping the Centre open on a one-to-one basis had been relatively successful, others described some problems such as the problem of limiting people in the Centre at any one time, and appointments being missed:

People will just turn up anyway. Some of them, because of the way their lifestyles are, they don’t stick for appointments. They turn up like two hours after you’ve arranged it. Because trying to keep staff members safe as well, so keeping social distancing guidelines. That has been a tough one for these folk really. (Caroline).

#### Balancing risks and benefits

Clients saw the Centre as a safe space and were keen to see that element restored, even in a restricted and adapted manner. The comfort and safety offered by the ‘drop in’ was described as providing a sense of home:

…to have that household atmosphere restored where people have a safe place for their friends with extra support if they need it. So even if you are homeless you do have this place that resembles a home. (Naomi, Stakeholder).

Staff thus discussed the ongoing challenge of balancing the uncertain level of risk posed by transmission of coronavirus against this need to provide a safe space, social contact and ongoing support. Staff described the safety measures they put in place such as: limiting numbers; enhanced cleaning; being strict about rules; and encouraging wearing of masks. Martin (Staff) discussed the importance of the Centre being assessed by public health professionals to ensure staff and Centre had the correct protocols if someone showed symptoms. Jack (Stakeholder) discussed an awareness amongst Centre staff and wider service providers that many clients using the Centre had underlying health conditions placing them in high-risk categories:

There is still a fear factor, there is a massive fear factor from, not us contracting it from the guys, it’s us giving it to the guys, because they are the ones with the underlying health issues. They have all got COPD and asthma and blood clots, DVTs [Deep vein thrombosis], alcoholism and drug use and so, they catch it, it could kill them, we catch it, 99% of us are going to come through it unscathed.

Whilst COVID-19 posed a severe risk for those using the Centre, the risks to clients through the closure of vital services were also described as grave. Whilst clients reported anger and confusion this was perhaps made more acute by a lack of initial understanding amongst some of the severity of risk posed by the virus, and of Government restrictions on people gathering together.

During the early stages of lockdown (see Additional file 4 for timeline), the Centre began to provide a hot meal to takeaway, in conjunction with food parcels, which became one of the main dilemmas regarding balancing risks. Provision of food initially was motivated by a concern that there may not be food available to those who did not have the budget or means to cook for themselves, but this needed to be weighed up against the risk of encouraging clients to gather in shared spaces. In order to mitigate risk of infection, markers were put on the ground outside the Centre, and people were encouraged to keep two metres away from each other. However, staff experienced challenges in ensuring clients kept their distance, raising concerns of *‘a virus hotspot’* (Kate, Staff), as clients gathered together to eat takeaway food and socialise:

People that we work with, for many reasons, really struggled to follow social distancing and take this on board… there is definitely that feeling of having very little regard for their own lives anyway, that there wasn’t that same sense of panic for them. And that was really tough. I found that really difficult… as a service that was based around relationships and building attachments and connections to then, all of a sudden, feel like you are almost re-traumatising people because you are saying ‘no, I can’t see you just now, you need to stand here’. It was just not how would we would normally communicate (Kate, Staff).

Staff used the opportunities of providing food parcels and hot food to also offer crisis intervention and emotional support. Martin (Staff) described this approach as *‘chaotic’,* due to the large queues out of the door, as well as emotionally difficult because of seeing people in distress and not being able to provide normal levels of care. However, by the end of April, there was a general consensus among the staff that the risks began to outweigh the benefits of continuing to offer food. Those accommodated in hotels had access to food, and other organisations in the city had started to fill this gap and provide food. Staff also worked with a charity organisation to provide fresh, ‘readymade’ meals to Bed and Breakfast accommodation, hostels, and single tenancies. The decision was therefore made to stop the takeaway food provision but continue to run a foodbank service, in collaboration with another service, and food deliveries.

#### The challenges of distanced communication

One of the major ongoing changes to Centre provision was the move to ‘distanced’ modes of communication, through: telephone support; online support groups to replace physical groups; and socially distanced one-to-one appointments. As described above, the online groups/telephone support were made possible by staff gathering client contact information and distributing smartphones. Staff also sat down on a one-to-one basis when distributing the telephones, showing clients the basics of how to operate them and access groups:

(Staff) were providing a lot of practical support over the phone, and emotional support too. Being able to maintain that connection with people, and knowing we were actively reaching out to engage, that that was a real positive for many people (Kate, Staff).

As individual situations became more settled over the course of lockdown, staff were able to move from crisis intervention to support focused on emotional and psychosocial needs. For Martin (Staff), the move to telephone support actually enabled deeper conversations about wellbeing than had occurred in the physical space of the Centre, because of the more systematic approach undertaken. Clients also regularly telephoned staff during specific times of need. While some challenges arose regarding telephone support, particularly when clients did not answer their telephones, all members of staff acknowledged that it was an important way of adapting support in very challenging circumstances.

The Centre’s provision of online support groups occurred fairly early in lockdown and developed as a result of feedback from clients that, whilst telephone support was vital, there was a need for more support. Groups included the regular psychosocial group, a women’s group, and a fitness group, which all ran either once or twice weekly. The online support also included a closed social media group for the women’s group where they could chat to one another. While there were some initial concerns that clients might struggle to use the technology required to access these online groups, the general consensus was that they had managed well, with groups being well attended. Some challenges were discussed regarding running these groups, however, with difficulties including background noise, ensuring clients observed boundaries, and comforting people who became agitated or upset.

Clients had a range of views concerning the Centre’s move to telephone support and online groups. All clients interviewed had received telephone support, although this was made difficult for a few by either not initially having a telephone, or because of losing their phone. However, the Centre seemed to have been able to maintain contact with these individuals. John described struggling with speaking on the telephone, leading to avoiding calls:

I’m a face-to-face kind of person. I struggle to pick up the phone as much as I probably could… When I do need to use it, I know that’s there, and I do pick up the phone when the going gets tough.

Maria described telephone support as ‘too robotic’, but said that receiving a telephone-call ‘breaks the fucking monotony’ of shielding.

### Scaling up harm reduction

As noted in introduction, harm reduction is a key feature of the Centre. Over the course of the pandemic, the provision of harm reduction services changed to reflect the ever-changing situation and needs of clients, including people who only started to engage with the Centre during the pandemic. The continued provision of harm reduction services over this time was beneficial to existing clients and those who had previously accessed other services which had closed. Participants talked about the central location of the Centre, and the nonmedicalised setting, as facilitating this engagement. The pandemic created opportunities to scale up harm reduction in three ways: an improved and internal IEP service; access to take home naloxone; and starting a multi-disciplinary health clinic within the Centre that provided a range of health care and substance use related services.

One of the initial changes involved moving the IEP service from the mobile van that visited weekly and parked directly outside into the Centre. At the beginning of the pandemic, it was clear that the van could not operate due to a lack of space for social distancing but that providing such a service was essential due to a lack of IEP services elsewhere in the city. This enhanced service was described as a ‘*one stop shop*’ (Richard, Stakeholder), providing essential harm reduction equipment within the context of supportive relationships. The second major development was being able to provide naloxone to clients, after years of trying to get permission to do so:

They’d been trying to get take home naloxone for ages. There were a lot of administrative [barriers]… we’d been pushing on that with them repeatedly. I kept thinking we’d resolved it and then it got blocked, and then resolved, and then blocked. And that had been going on for six months or maybe a year. There were legal barriers, there were financing barriers, there were all sorts. And what happened a week after the COVID crisis kicked in? I said ‘Oh for God’s sake what now? Are you still going to keep squabbling about this?’ So that went through very quickly. (Richard, Stakeholder).

It took a global pandemic to address these barriers and, currently, such naloxone provision is only permitted for the duration of the pandemic, although several participants highlighted the need for this to be made permanent given the concurrent drug-related death public health emergency Scotland is also experiencing. Staff created a poster during the pandemic to encourage naloxone use and address stigma, which was also worn on clothing (see Additional file 5).

The third important change that occurred as a scale up of harm reduction in the Centre, as a result of the pandemic, was the introduction of an enhanced OST service offering same day prescribing and titration (gradually increasing medication dosage over a period of days and weeks, ideally until an optimal dose is achieved) within a new multi-disciplinary health clinic that started operating in the Centre on the 1st April. While this proposal was submitted just prior to the pandemic, and initially approved, it was then rejected by NHS managers. However, with the onset of COVID-19 and subsequent suspension of many other city services, the proposal was then approved. Despite the confusion and concerns about transmission risks, the pandemic was therefore viewed by many participants as providing real opportunities for enhanced harm reduction within the Centre, as Richard (Stakeholder) described:

The drop-in availability for titration which is brilliant, and another positive effect of the COVID situation. A very stupid administrative barrier got removed. The harm reduction values that they were starting with have been very well reinforced by the situation. I mean they already operated like it was a crisis because it always has been a crisis. And that is a harm reduction instinct… of dealing with the reality of where people are, and what they need. So it’s reinforced the value of a lot of what they already did, philosophically and practically. They built relationships, they networked, they look out to research and to innovation, and they seem to make decisions well and quickly.

Both staff and stakeholders were concerned about the longevity of these changes and wanted to ensure they would continue post-COVID-19. Relatedly, there were concerns about a lack of more sustainable streams of funding for some of these developments:

We have no idea if there will be ongoing support for this going forward… once the pressure of the pandemic is off. It’s really tricky to know if generic services are going to value this and want to keep supporting it. (Max, Stakeholder).

Finally, several participants noted that there was a marked difference in the provision of harm reduction services for people who predominantly used drugs, compared to alcohol. Much of the enhanced service was related to the provision of injecting equipment, naloxone, and rapid access to OST, with alcohol harm reduction approaches notably lacking, as Martin (Staff) highlighted:

There has been a lot of stuff in terms of drug misuse, so the needle exchange, you’ve got the replacement therapies, they have got naloxone but actually, if alcohol is your problem, we didn’t have very much. That’s been the big gap (…) because there are a few guys that have really been struggling with alcohol. (…) Alcohol needs to come back to the fore.

One of the major challenges during the early period related to staff having to adjust to new ways of working. Staff discussed concerns in terms of working from home, whether they would have enough equipment, the need for more specialist knowledge regarding harm reduction interventions, and how best to support each other and clients. To address these concerns, additional training was provided, for example on OST and IEP and, initially, weekly online reflective practice sessions were run to provide staff with additional support opportunities. Max (Stakeholder) reflected that being a third sector organisation, rather than a statutory service, allowed the Centre to be flexible and adapt and learn as they went along. One staff member felt that the Centre’s flexibility was the result of not being contracted externally and therefore required to meet certain criteria. Internal funding (The Salvation Army funds all service provided at this Centre) allowed the service to adapt and flex to meet the changing needs presented to them.

Supporting staff with the emotional toll of providing distanced support was compounded by the need for some to be working from home, and to balance work and family life:

Working from home during this time was a real challenge because staff also have families and they’re managing that. And providing a new way of supporting people over the phone is completely different. To make sure staff felt supported enough we created a WhatsApp group to stay connected and that was all really great in the beginning, and increased supervision to weekly. (Kate, Staff).

Staff commented on the positive team dynamic, effective communication, and provision of mutual emotional support, where the team ‘*pulled together*’ (Caroline, Staff) to navigate uncharted territory. Strong and supportive management was highlighted as being key to facilitating this strong teamwork.

### What difference did it make?

Amongst clients, despite the tremendous challenges experienced during this period, there was a general perception that the Centre had been a vital source of support: a ‘life-line’. All interviewed clients had maintained contact during the lockdown, albeit sporadically in a couple of cases. Several discussed the Centre being their primary, or in some cases only, source of support during the pandemic. They discussed receiving food parcels and prescriptions delivered to them which were beneficial due to shielding and financial hardship. Several participants talked about the provision of harm reduction, such as the IEP service coming inside the building, as showing that the Centre cared about their wellbeing. A number of clients discussed struggling to engage with other services, most notably mainstream health services, stating that the online groups were a valuable source of support to support their mental health and combat social isolation. Steven (Client) discussed how, despite his fear, he had engaged with this form of group support, explaining that it had helped him ‘proceed the way I believe that I want to be as a person’, while other services had refused to support him:

Once you’ve made a mistake, and you’ve upset the boundaries of the rules and regulations, they tend to just never let you back in their lives ever you know. And they always say it’s because they are busy trying to fix someone else, someone else could use that time. And yet they dinnae think of the future.

Even for those who either struggled, or chose not to engage with wider supports available at the Centre, the safe space and one-to-one support was described as invaluable:

A support worker for someone who hasn’t got nothing anywhere else is very important. It means at least you are not falling through the cracks which happens with a lot of people. (Andrew).

I mean it from the bottom of my heart [Centre staff] are the only people that has been helping me over the last few months and it’s keeping me going. (Frank).

Clients discussed their desire for positive change in their lives and connected these hopes and desires with the support provided, including close and trusting relationships with staff:

I love each and every one of yous, I really do. I cannae imagine my life without any of yous, especially without some of you, ken, and just with the support that I get ken, although I get it from everybody I know who to go to for what. (Jacqui).

Staff with lived experience of homelessness were described as particular sources of inspiration for clients to improve their own circumstances. In addition to the desire to reduce use of drugs, alcohol, and substitute medication, clients discussed attaining an improved quality of life including: regaining care of children; improving health through exercise; gaining weight; practicing hobbies; and developing skills in educational, vocational, and employment-related activities. Throughout interviews the support offered by the Centre appeared to be crucial in helping individuals both imagine and enact such changes, though some discussed a fear of letting staff down:

I was struggling, I wouldn’t do it, because I felt I would just let yous down and I didn’t want to do something to encourage that. You see good progress in me and then, a blink of an eye, because I relapse on alcohol and just ruin it all, do you know what I mean? It hurts me… I’ve been doing it all my life. (Steven).

Planning to make both simple yet often far-reaching changes could all be done in a safe and homely environment, which facilitated people becoming more compassionate and kind towards themselves. A number of clients had volunteered for the Centre in the period prior to COVID-19. Such volunteering provided opportunities to get involved, as well as improving daily structure, a sense of ownership of the Centre, and an opportunity to develop skills and knowledge:

I really enjoyed doing the needle exchange and having the opportunity to also go out and help the workers to be safe at night. I got a right… what’s the word? I got a right sense of self-worth. (Jacqui).

As demonstrated, the Centre’s approach was to treat clients with patience and respect at all times, and allow individuals to engage at their own pace.

### Change, change, and change again: a ‘whole new life’

A recurring theme throughout the interviews was a sense that the pandemic, and the Centre’s response, was a learning curve that no one had ever faced before, and that the need for constant change would continue, even as lockdown measures eased. Kate (Staff) discussed the difficulty of constant change, and a desire amongst staff for a more settled period to ‘steady the ship’. A number of participants discussed the Centre’s adaptability and flexibility as key to its ability to continue delivering crucial services in such circumstances. For one stakeholder, the period of change which the Centre had undergone pre-COVID-19 was crucial for instilling an organisational culture open to adaptation and change. Some highlighted the ‘levelling’ impact of the pandemic:

All of us are in the same boat, none of us have lived with this type of pandemic before. We’re all daily or weekly or hourly figuring out different ways to do it. A lot of head scratching, soul searching, all of the right ways. (Jack, Stakeholder).

Another stakeholder expressed concern that the Centre may not return to what it was before the pandemic, as a result of social distancing likely becoming long term. Many had the perception that the pandemic would impact the city service landscape in long-lasting ways. Frank (Client) summed this up as *‘a whole new life’*. For Kate (Staff), the opportunity to get client feedback in a more systematic way was key to adapting well to this tumultuous period:

My feeling now is that it’s going to be some time before the Centre is how people experienced it before. So it’s really important that the individuals we work with feel that they are involved in any future changes.

This process of gaining client feedback was ongoing through the pandemic and continues. Regarding COVID-19 service recommendations, the online groups were valued for the longer-term, plus provision of additional outreach-type support for people who were shielding (to their own accommodation) to reduce social isolation. Clients also made a range of general service recommendations, including extending the length of the psychosocial group sessions; a group specifically for those in recovery from problem substance use; a men’s group; education, employment and skills training groups; changes to the rules and physical space of the Centre; allowing dogs; extending opening hours including into weekends; and running more social activities both internal and external to the Centre. Despite online and telephone support being highly valued, clients appreciated and missed the comfort and safety of the physical space provided by the Centre, and the practical support received which, they believed, was unable to be fully replicated via telephone, online, or even one-to-one appointments.

## Discussion

Overall, clients perceived the Wellbeing Centre as having met their needs during the pandemic. Although there were obvious limits imposed on this, and some still struggled daily with social isolation, mental health problems and substance use, alongside a wealth of challenges including just surviving, clients perceived the adapted services offered as a ‘lifeline’. The ongoing support/services offered included: distribution of telephones with data; provision of telephone/online support; supporting assertive outreach teams to access clients in need; ensuring clients had appropriate accommodation, were able to receive/pick up prescriptions and access food; providing a foodbank; one-to-one appointments with clients; running an IEP (see list of abbreviations in Additional file 6) and harm reduction service within the Centre; distribution of naloxone; and facilitating operation of a multi-disciplinary health outreach clinic which included rapid and regular access to OST.

Those who used the Centre reported a range of complex challenges that are consistent with the literature on deep exclusion and multiple intersecting physical and mental health problems, including  substance use concerns [53]. These challenges are compounded by ongoing complex trauma, discrimination, poverty, and pervasive instability. The COVID-19 pandemic placed significant additional stressors, particularly in the first few weeks of the lockdown in Scotland. In these weeks clients described confusion, loss, and isolation. Many of their services closed. Faced with the complex and varied needs of their client group, the Wellbeing Centre stayed ‘open’, rapidly changing what they offered and how, and continuing to change through the months of the lockdown. All clients who participated in the study maintained contact with the Centre during the pandemic, although this was made challenging for a couple who did not manage to keep their telephones. The support offered over these days, weeks, and months, offered an opportunity for individuals to feel valued, engage in constructive activities and support on their own terms. This impacted positively on people’s identity and lives, despite the wider uncertainty of that time.

Wider literature demonstrates that those who are homeless, or at risk of homelessness, have typically experienced much trauma, including violence, and severe hardship, and thus struggle to form trusting relationships [54, 55]. The Centre clearly operates a psychologically informed environments (PIEs)-informed approach, providing clients with a warm, friendly service. Value is placed on building trust and relationships, with clients describing feeling cared for and safe, even when they were unable to access the Centre in person or in the usual way via drop in style support. There is a clear sense of ownership of the Centre from clients and a culture of community where the Centre was described unambiguously as *their* service. Participants discussed the Centre’s ‘elastic tolerance’, as opposed to the sometimes punitive approach taken to them elsewhere: a key component of PIEs. Clients viewed the service as a means of meeting their psychological needs through safe space, dialogue, psychosocial group work, women-only spaces, and volunteering opportunities. A pro-active stance towards wider partnership working across the city also fits with the PIEs approach [56]. .

The support provided represented more than a discrete bundle of services aimed at meeting different needs such as substance use, health, housing, food, practical support, social connections, and harm reduction support. It is clear from client descriptions that the Centre has succeeded in moving relationships beyond that of care provider and recipient. Instead, it is perceived as a community space of love, care and safety, which allows clients to identify their own important goals and support needs, and move towards them. Clients’ descriptions of engaging with the service through volunteering, training, and introducing new attendees to the service, demonstrate that the Centre seeks not only to address clients’ problems and support needs, but also to actively build on their strengths. As Duff [42]; p. 207] has highlighted, harm reduction may become reduced to a ‘sterile policy prescription’ if there are no everyday interactions, displays of care, or elements of reciprocity with others [42]; p. 208]. For Duff, such co-produced, flexible and holistic models of harm reduction create ‘cultures of care’. The Centre has succeeded in creating a culture of care, as an innately relational, flexible service which allows clients to gain a sense of self-efficacy and also, via partnership working, provide a natural linking point into wider city resources.

The importance of ‘place’ was a central finding of the research where the Centre was described as a place of care and support. Spaces of care can have the ‘capacity to tend to isolation, stigma, shame, marginalisation, fear, pain and anxiety in uniquely caring ways’ [57]; p. 215]. The ‘care’ which constituted the Centre space was described as producing feelings of safety, positive identity, love, and the potential for improved life circumstances. Such ‘broader enactments of care’, which are innately relational, would be missed by a view which conceived of ‘care’ and ‘place’ as static or fixed. The changes that took place within the Centre due to the COVID-19 pandemic evidences Duff’s contention that ‘care’ and ‘place’ are fluid concepts constructed through networks of relationships and practices [42]. While the crucially important physical space of the Centre (drop in, café) was forced to close for large groups, the ‘place’ was then extremely rapidly reconstituted as one of ‘distanced’ care, consisting of online groups, telephone support, and socially distanced appointments. This loss of care via the usual methods was profound and acutely felt by both clients and staff. However, owing to the dense network of ‘relational’, ‘affective’, and ‘social’ aspects of ‘place’ and ‘care’ which staff had built with those that used the Centre, the service was able to flex and adapt to continue to be a ‘life-line’ during these unprecedented days and weeks. In a range of ways it managed to consolidate and intensify support in some important ways, including scaling up its harm reduction services, and offering a vital component of Edinburgh city centre’s COVID-19 response to people experiencing homelessness.

Staff discussed the challenges that they experienced related to supporting vulnerable people from a distance and managing the constant worry about their wellbeing. These are some of the well-documented emotional and psychological difficulties of working with people with complex lives, including homelessness [58]. Those providing support to such individuals are placed in chaotic and challenging situations on a daily basis, with high rates of burnout and high staff turnover [59], which also impacts on the service able to be provided and client experiences of support [60]. Reflective practice, supervision, staff meetings, and effective training, can have positive effects on staff wellbeing [58], and are also critical within a PIEs-informed approach [45]. Organisational culture and leadership are key to ensuring staff are well supported in their roles, and the current COVID-19 pandemic makes staff support even more vital. Training has been a significant feature of the Centre, including on topics such as emotional regulation, psychosocial support, and harm reduction. This investment in training and staff support played an important role in creating a strong team dynamic that was able to rise to the challenge presented both personally and professionally during the pandemic. They were able to navigate the uncertainty of the situation together, support one another, and continue to provide emotional and practical support to their clients.

### Strengths and limitations of the study

This study provided insight into one third sector service in Edinburgh, Scotland, at a time of great upheaval, capturing service changes as they were happening. Due to an existing strong partnership between researchers and the service organisation, we were able to conduct in person socially distanced data collection with people using services during the height of the pandemic, something that was not feasible for other researchers. The use of fieldnotes and reflexivity ensured that researcher bias was actively taken account of throughout. Use of different participant viewpoints provides a rich picture of this very intense and demanding period. The client sample was largely pragmatic i.e. those who were actively engaged with the service, who may therefore have held more positive views of service provision. We also used purposive sampling across all participant samples to try to reduce undue bias [61]. By assuring participants of the voluntary nature of participation, we believe participants were able to be open about their experiences and discuss both positive and negative aspects of the Centre, including suggested improvements. Relatedly, all client interviews were conducted by a community/peer researcher, who also had a role in the service as a peer support worker (JD). Their dual role may also have led to positive response bias and the inability of clients to make more negative comments. In anticipation of this, all clients were offered the opportunity to be interviewed by a university-based researcher, unconnected to the service, but none took up this option. The view of our research team is that the existing honest and open relationship between the community researcher/peer support worker and those using services helped to facilitate our study’s in-depth interviews, with rich descriptions of extreme challenges and the role that the Centre played in people’s lives. As reflected in the data, participants did seem to be comfortable in being critical or discussing their concerns about the changes that took place in the Centre. By providing such critical comments, opportunities were created for this feedback to contribute to further developments of the Centre, some of which have now been operationalised (November 2020). Providing feedback regularly was something clients were accustomed to.

### Implications for policy and practice

Study findings demonstrate the benefits of rapid access to OST, enhanced provision of IEP, and provision of naloxone, for those experiencing homelessness and problem substance use, within third sector services. Such service provision should continue because of the effectiveness of these interventions in reducing harms such as BBVs, and drug overdose, in this population [62]. More attention needs to be focused on alcohol harm reduction which continues to be neglected [60]. Removing policy barriers, for example to naloxone provision to people at risk of overdose, is essential post-COVID-19. Importantly, telephone and online provision of support was demonstrated to be both feasible and acceptable for this population and should therefore continue, alongside in person practical support, where it is safe to do so and following public health and government guidance. The new support communication methods that were trialled under severe pressure within this service have shown the potential for providers and clients to keep in touch through a pandemic, and demonstrate care and kindness, when social distancing is required to keep people safe. The provision of smartphones can also enhance digital literacy. Holistic and flexible models of care and support, allowing individuals to engage on their own terms, build trust and long-lasting relationships that can ‘flex’ when challenged to meet new demands. Staff working in third sector homelessness services need support and good leadership, and opportunities for training, support and reflective practice, which should continue despite the pressures of providing an enhanced service to a client group. To ignore their needs risks compassion fatigue and burn out. Partnership working between services is also essential, and the COVID-19 pandemic has highlighted the essential role that this plays within a treatment and service system. Finally, the rapid rehousing of people who are homeless was likely an essential element of the care and support provided during this time. Teixeira (2020) has provided a challenge to the homelessness sector and beyond: ‘does this crisis shift what we think is possible, conceivable or ‘normal’?’ [14]. Our findings demonstrate that yes, indeed, this study has shown how much more can be done to provide holistic, responsive, harm reducing, and health protecting, support to people who are homeless.

#### Conclusion

The aim of this novel study was to explore how one third sector service for people who are homeless, or at risk of homelessness, responded to the pandemic. Very few studies, to date, have focused on this population and sector. It provides a unique insight into the pandemic response by collecting and triangulating data from clients, staff, and wider stakeholder professionals, during the height of the pandemic in Scotland, including socially distanced in person interviews with those using services and service documents. The study has provided rich description regarding the loss, confusion and isolation experienced by people who were homeless, and the essential support provided to them when many other services literally closed their doors. Practical and emotional support was continued using new methods such as telephone and online groups, and harm reduction services were successfully scaled up, providing much needed support for people who use drugs at risk of overdose and a range of further harms. Those using the Centre described the stability that this created for them during a period of profound disruption and insecurity. There are many implications for policy and practice that have international relevance for service commissioners and providers, and those designing national policy for responding to the COVID-19 pandemic, including: the need for continued easy access to harm reduction approaches for both drugs and alcohol; the provision of telephone and online support, with smartphones being provided for those who need them; and, importantly, the need for continued support, training, and reflective practice for staff working in these services.

## Supplementary Information

**Additional file 1.** Full study research questions.

**Additional file 2.** Interview schedules for all participants.

**Additional file 3.** Glossary of Scottish dialect.

**Additional file 4.** Timeline of changes that occurred in the Centre.

**Additional file 5.** Poster created by staff to encourage naloxone use and address stigma.

**Additional file 6.** List of abbreviations.

## Data Availability

The datasets generated and/or analysed during the current study are not publicly available because study data were generated to evaluate the response of one small service to the COVID-19 pandemic. Individual privacy could be compromised if the dataset is shared due to the small sample involved.
