# Supplementary material for: ‘They already operated like it was a crisis, because it always has been a crisis’: a qualitative exploration of the response of one homeless service in Scotland to the COVID-19 pandemic
Source: Harm Reduct J. 2021 Mar 3;18:26. doi: 10.1186/s12954-021-00472-w (PMC7927775; doi:10.1186/s12954-021-00472-w)
Supplement: Supplementary file 1 — Additional file 1. Full study research questions. [file 12954_2021_472_MOESM1_ESM.docx]

**Additional File 1. Study Research Questions**

The study research questions were:

1) How did client needs change in the early days and weeks of the Covid-19 pandemic?

2) What was already in place in the Wellbeing Centre to meet the needs of clients?

3) What changes and adaptations have been implemented since the start of the Covid-19 pandemic for those experiencing homelessness/risks of homelessness and/or substance dependencies?

4) What opportunities and benefits have been presented by Covid-19? What challenges, barriers, and risks have been identified for clients, for the service as a whole, and for staff members, and in relation to service adaptations?

5) What barriers continue to be a problem in terms of meeting the needs of people most vulnerable?

6) What lessons can be learned for the service and wider, and how can benefits be maintained and any risks mitigated?
