# Supplementary material for: ‘They already operated like it was a crisis, because it always has been a crisis’: a qualitative exploration of the response of one homeless service in Scotland to the COVID-19 pandemic
Source: Harm Reduct J. 2021 Mar 3;18:26. doi: 10.1186/s12954-021-00472-w (PMC7927775; doi:10.1186/s12954-021-00472-w)
Supplement: Supplementary file 2 — Additional file 2. Interview schedules for all participants. [file 12954_2021_472_MOESM2_ESM.docx]

**Additional File 2. Interview schedule for face-to-face interviews with clients**

1. What services were available to you at the Wellbeing Centre in the six months prior to Covid-19?

2. When did you first realise changes were coming to the Wellbeing Centre in response to the Covid-19 pandemic? *Timeline – pandemic was announced by WHO on 11^th^ March; UK lockdown started 23^rd^ March.*

3. What ongoing changes have there been at the Wellbeing Centre during Covid-19?

4. What, if anything, would you keep from these new ways of working once lockdown restrictions are lifted?

5. If you had to sum up the Wellbeing Centre in three words, what would they be?

6. Finally, is there anything else that you would like to add?
