# Supplementary material for: ‘They already operated like it was a crisis, because it always has been a crisis’: a qualitative exploration of the response of one homeless service in Scotland to the COVID-19 pandemic
Source: Harm Reduct J. 2021 Mar 3;18:26. doi: 10.1186/s12954-021-00472-w (PMC7927775; doi:10.1186/s12954-021-00472-w)
Supplement: Supplementary file 3 — Additional file 3. Glossary of Scottish dialect. [file 12954_2021_472_MOESM3_ESM.docx]

**Additional File 3. Glossary of Scottish terms**

Ain - own

Cannae – can’t

Dae – do

Didnae – didn’t

Dinnae – don’t

Fae - from

Gi’ – give

Gonnae – going to/gonna

Havenae – haven’t

Isnae – isn’t

Ken – know

Mare - more

Nae – no

Naebody – nobody

Naewhere – nowhere

No – not

Oot - out

Tae – to

Wasnae – wasn’t

Wee – small/little

Wi’ – with

Wouldnae – wouldn’t

Yous – you (plural)
