# Supplementary material for: ‘They already operated like it was a crisis, because it always has been a crisis’: a qualitative exploration of the response of one homeless service in Scotland to the COVID-19 pandemic
Source: Harm Reduct J. 2021 Mar 3;18:26. doi: 10.1186/s12954-021-00472-w (PMC7927775; doi:10.1186/s12954-021-00472-w)
Supplement: Supplementary file 4 — Additional file 4. Timeline of changes that occurred in the Centre. [file 12954_2021_472_MOESM4_ESM.docx]

**Additional File 4. Timeline of service changes**
