# Supplementary material for: ‘They already operated like it was a crisis, because it always has been a crisis’: a qualitative exploration of the response of one homeless service in Scotland to the COVID-19 pandemic
Source: Harm Reduct J. 2021 Mar 3;18:26. doi: 10.1186/s12954-021-00472-w (PMC7927775; doi:10.1186/s12954-021-00472-w)
Supplement: Supplementary file 5 — Additional file 5. Poster created by staff to encourage naloxone use and address stigma. [file 12954_2021_472_MOESM5_ESM.docx]

**Additional File 5. Naloxone poster used in the service**


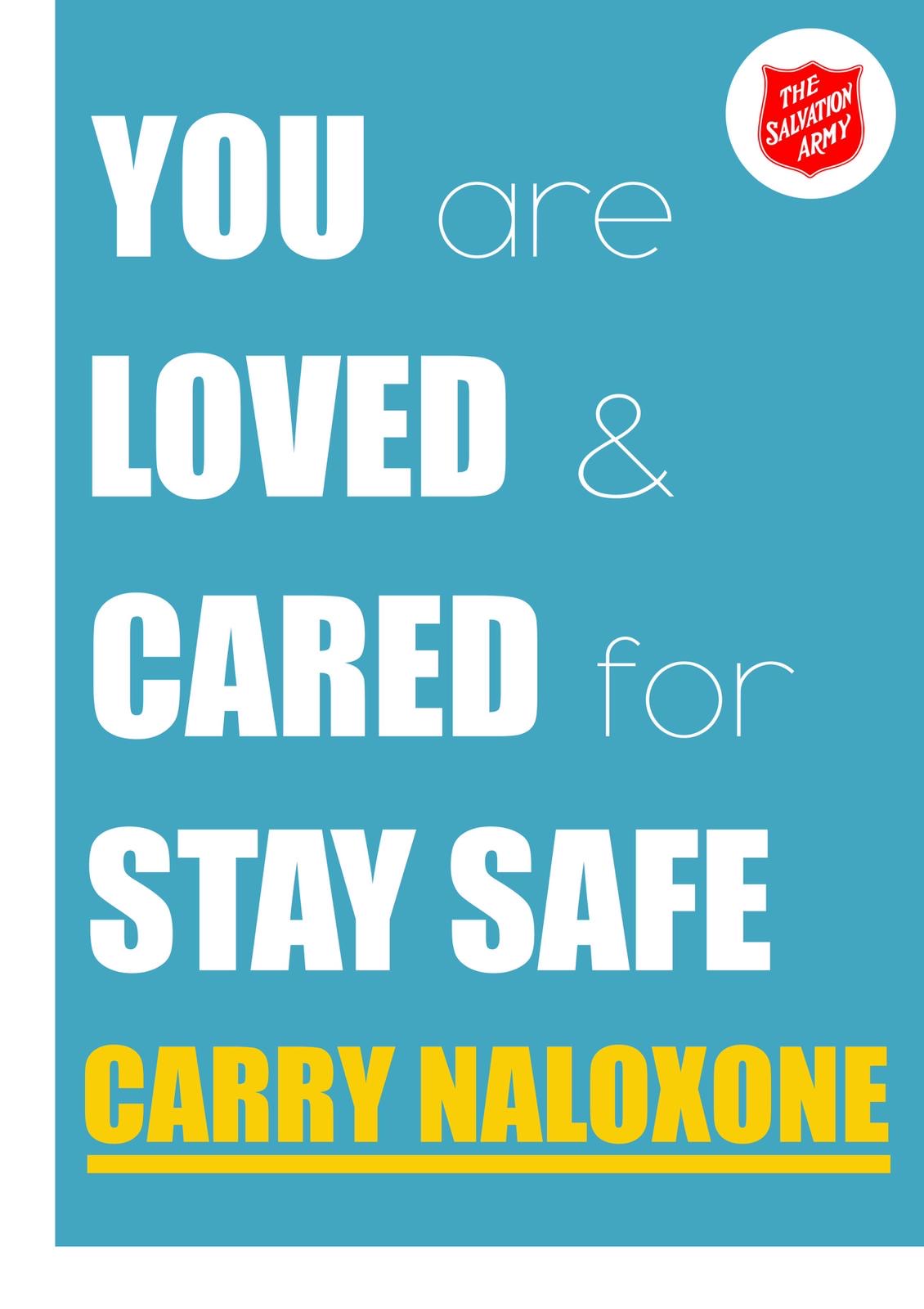


This poster was created by staff in the Wellbeing Centre and designed by Rosa Loves Rainbows. Permission has been granted by the Wellbeing Centre to use the image in this paper.
