# Supplementary material for: ‘They already operated like it was a crisis, because it always has been a crisis’: a qualitative exploration of the response of one homeless service in Scotland to the COVID-19 pandemic
Source: Harm Reduct J. 2021 Mar 3;18:26. doi: 10.1186/s12954-021-00472-w (PMC7927775; doi:10.1186/s12954-021-00472-w)
Supplement: Supplementary file 6 — Additional file 6. List of abbreviations. [file 12954_2021_472_MOESM6_ESM.docx]

**Additional File 6. List of abbreviations**

BBV - blood-borne virus

COPD - chronic obstructive pulmonary disease

CPN - Community Psychiatric Nurse

DVT - deep vein thrombosis

FTE - full time equivalent

IEP - injecting equipment provision

MAP(s) - Managed Alcohol Programme(s)

NHS - National Health Service

OST - opioid substitution treatment

PIEs - psychologically informed environments

PPE - personal protective equipment

SHAAP - Scottish Health Action on Alcohol Problems
